# Nova Pneumonia por Coronavírus e Miocardiopatia: Relato de Caso

**DOI:** 10.36660/abc.20200268

**Published:** 2020-05-22

**Authors:** Mustafa Ahmet Huyut

**Affiliations:** 1 Yeni Yuzyil University Istanbul Turquia Yeni Yuzyil University – Cardiology,Istanbul – Turquia

**Keywords:** Doenças Cardiovasculares/complicações, Coronavirus, COVID-19, Miocardite, Cardiomiopatias, Doenças Infecciosas, Síndrome Respiratória Aguda, Pneumonia, Ecocardiografia/métodos, Tomografia Computadorizada/métodos, Hospitalização

Um agrupamento de casos de pneumonia foi registrado pela primeira vez em Wuhan, Hubei, China, em dezembro de 2019.^1^ Foi estabelecido que um coronavírus era o patógeno responsável pela doença e, desde então, é chamado de Coronavírus da Síndrome Respiratória Aguda Grave 2 (SARS-CoV-2). A doença desencadeada pela SARS-CoV-2 é chamada COVID-19, que se espalhou pelo mundo desde então. Os números tendem a subir na Europa e a extensão da letalidade da COVID-19 não pode ser medida corretamente. Em pacientes idosos, a letalidade parece ser particularmente maior quando comparada à influenza sazonal.^2^ A TC é muito útil no diagnóstico da COVID-19.^3^Além disso, o exame de ecocardiografia transtorácica (ETT) é uma ferramenta muito importante para avaliar a fração de ejeção do ventrículo esquerdo (FEVE). Estudos anteriores relataram que a reação em cadeia da polimerase em tempo real (RT-PCR) era o atual padrão ouro para o diagnóstico de COVID-19.^3^ Mas, em alguns casos, a sensibilidade da TC é maior que a da RT-PCR.^4^ Relatamos um caso confirmado de pneumonia por COVID-19 em uma mulher de 59 anos. Encontramos uma diminuição leve da FEVE sem elevação dos níveis da troponina-I, que pode ser considerada como miocardiopatia devido ao aumento da liberação de citocinas na COVID-19. Até onde sabemos, este é o primeiro relato da literatura que demonstra a associação entre imagens de ETT e TC na COVID-19, e descobrimos que a piora nos achados da ETT está alinhada com a progressão das imagens na TC.

## Relato de Caso

**Figura 1:**
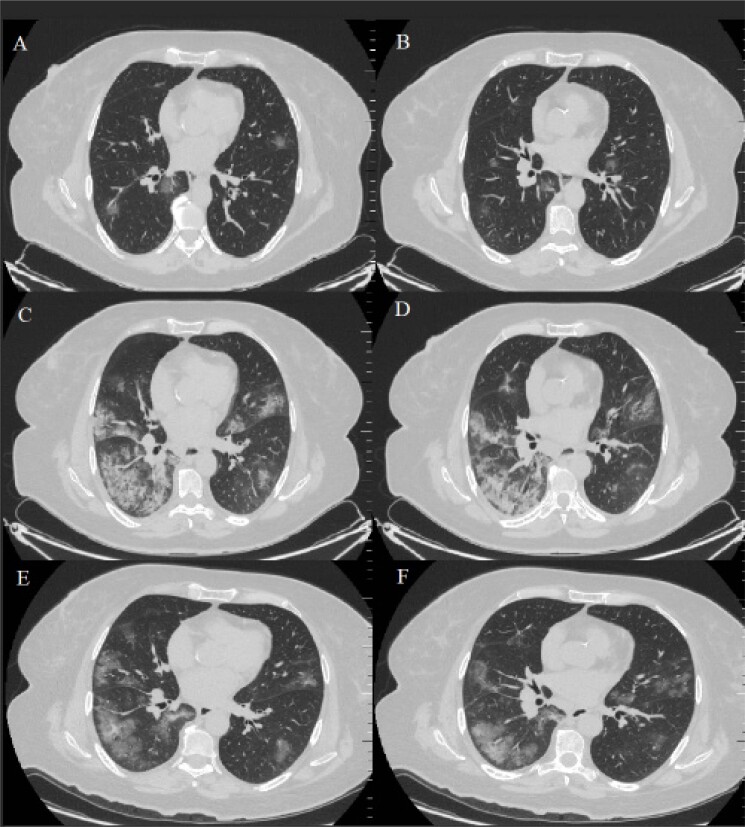
– *Imagens axiais de TC. **(A-B)**: Na hospitalização, a TC mostra a presença bilateral de GGO leve no parênquima. **(C-D)**: No dia 6, uma TC de repetição foi consistente com a crescente expansão dos GGOs e com as consolidações em andamento, chamadas de “pavimentação em mosaico”. **(E-F)**: No dia 12, uma TC de repetição mostrou que as consolidações anteriores e GGOs em ambos os pulmões tinham sido absorvidos em sua maioria, deixando lesões fibrosas que podem indicar pneumonia residual em organização. TC: tomografia computadorizada, GGOs: opacidades em vidro fosco.*

Uma mulher de 59 anos teve febre por 4 dias, depois de pegar um resfriado. Um dia antes de visitar o hospital, ela estava com febre e tosse, mas sem sensação de aperto no peito, dor no tórax, calafrios, náusea e vômito ou diarreia. Ela não se sentiu melhor depois de receber antitérmicos. Então, ela foi internada em nosso ambulatório no BHT Clinic Tema Hospital. Quatro dias antes, a paciente havia tido contato com um parente que havia viajado pela Europa. Em seu histórico médico anterior, cirurgia bariátrica havia sido realizada três anos antes e ela ainda apresentava diabetes mellitus tipo II, hiperlipidemia e hipertensão como condições pré-existentes. Ela foi internada em nosso hospital em 20 de março de 2020 e ainda apresentava febre após a internação, com a temperatura mais alta de 39,5 °C, frequência cardíaca de 119 batimentos por minuto; a eletrocardiografia foi consistente com taquicardia sinusal e o QTc foi calculado em 0,398 segundos, pressão arterial em 94/60 mmHg e taquipneia conspícua com frequência respiratória de 24 incursões respiratórias/min com oxigenação suficiente (95% de saturação em ar ambiente). A identificação do novo coronavírus em 2019 (2019-nCoV) na RT-PCR foi positiva em uma amostra obtida com um *swab* de garganta. O risco de contaminação simultânea com outros vírus respiratórios e outros patógenos foi negativo para a amostra obtida do *swab* de garganta. As características tomográficas da paciente foram semelhantes às séries de casos relatadas por Pan et al.,^5^ (Figura 1: A-B). Os resultados laboratoriais mostraram leucopenia, com 4,1 × 10^9^/L, linfopenia com 0,8 × 10^9^ / L, nível apenas ligeiramente aumentado de PCR, com 18,4 mg / L e baixo nível de procalcitonina, com 0,01 ng / mL. A paciente foi submetida a um exame de ETT com transdutor de 3,5 MHz (Vivid-7 GE Medical System, Horten, Noruega). Os exames e medidas foram realizados de acordo com as recomendações da *American Echocardiography Unit*. O método de Simpson foi utilizado para calcular a FEVE.^6^ Na admissão, a FEVE foi calculada em 65%, com achados normais de ETT.

A paciente foi isolada e iniciou terapia de alto fluxo nasal para insuficiência respiratória e tratada com medicamento antiviral (oseltamivir, 75mg / cápsula, 1 cápsula por vez, duas vezes ao dia, por 5 dias), antibiótico (azitromicina, 500mg / comprimido) no primeiro dia e depois 250mg / comprimido, uma vez ao dia por 4 dias), antipirético (paracetamol 1gr / 100 mL, duas vezes ao dia), mucolítico (ampola de N-acetilcisteína, 300mg / 3mL intravenosa (IV), duas vezes por dia), anticoagulante (enoxaparina 4000 anti-Xa UI / 0,4 mL, uma vez ao dia), corticosteroide (metilprednisolona, 40mg intravenosa (IV), uma vez ao dia, por 5 dias), inibidor da bomba de prótons (ampola de esomeprazol, 40 mg IV, uma vez ao dia) e medicamento antimalárico (sulfato de hidroxicloroquina 200 mg / comprimido, 400 mg / comprimido duas vezes ao dia no primeiro dia e depois 200mg / comprimido duas vezes ao dia, por 6 dias).

Após 5 dias de tratamento, a temperatura da paciente voltou ao normal e os sintomas desapareceram. No entanto, no dia 6, uma TC de repetição mostrou-se consistente com o aumento da expansão dos GGOs e progrediu para as chamadas consolidações de “pavimentação em mosaico”. (Figura 1: C-D). Além disso, a FEVE foi calculada em 52%, mas o nível de troponina-I ainda era normal. Devido aos resultados da TC e aos achados da ETT, adicionamos favipiravir ao tratamento (200 mg / comprimido no primeiro dia, 1600 mg / comprimido duas vezes ao dia e 600 mg / comprimido duas vezes ao dia por 4 dias) em vez do oseltamivir. No 12º dia, uma TC de repetição mostrou que as consolidações anteriores e GGOs em ambos os pulmões tinham sido absorvidos em sua maioria, deixando algumas lesões fibrosas que podem indicar pneumonia residual em organização (Figura 1: E-F). Além disso, a FEVE foi calculada em 65% e a repetição da RT-PCR foi negativa e a paciente recebeu alta. Nenhum outro exame tomográfico de acompanhamento foi realizado.

A infecção é transmitida principalmente através de gotículas respiratórias. Febre e tosse seca são os principais sinais clínicos da COVID-19 em pacientes, acompanhados de dores no corpo ou exaustão, e a maioria dos pacientes tinha entre 40 e 60 anos de idade. Além disso, em alguns casos, podem ocorrer dor de cabeça, hemoptise e diarreia. Além do mais, pacientes graves podem evoluir para SDRA e a intubação pode ser necessária em alguns pacientes.^1^ Os sinais clínicos da COVID-19 são os mesmos de infecções normais do trato respiratório superior, mas a TC do tórax mostra alguns detalhes.^5^ Entretanto, é difícil distinguir o COVID-19 de outras pneumonias virais com base apenas nos achados da TC. Ainda é necessário esclarecer e definir a história epidemiológica e ela deve ser diagnosticada por RT-PCR. Miocardite aguda é um risco documentado de infecções virais, tais como a influenza. A apresentação clínica varia de miocardite assintomática a fulminante, o que pode contribuir para instabilidade hemodinâmica grave.^7^ Estudos anteriores baseados nas autópsias em casos fatais mostraram que, durante a pandemia de influenza asiática de 1957 e durante a pandemia de influenza espanhola, foram registradas, respectivamente, 39,4% e 48% de taxas de complicações com miocardite focal a difusa.^8^ Esses incidentes mortais de miocardite mostraram pneumonia grave e envolvimento de múltiplos órgãos. Como consequência, espera-se que a miocardite seja um risco fatal em um surto pandêmico de influenza. Miura et al.,^9^ também encontraram um antígeno viral no miocárdio com coloração imuno-histoquímica do coração autopsiado.^9^ Bowles et al.,^10^ avaliaram amostras de biópsia endomiocárdica de 624 pacientes e identificaram objetivamente miocardite utilizando PCR para diferentes genes virais. Das 239 amostras positivas para genes virais, o adenovírus foi encontrado em 142 amostras, o enterovírus em 85 amostras e o influenza tipo A em apenas cinco (0,8%) amostras.^10^Portanto, embora a patogênese da miocardiopatia ou miocardite associada à COVID-19 permaneça incerta, a literatura sugere que a disfunção endotelial pode ter um papel importante na patogênese da miocardite e da miocardiopatia. Os achados de análises por microscópico eletrônico do coração a partir de um modelo murino de miocardite por influenza mostraram muitos linfócitos infiltrantes diretamente ligados aos miócitos cardíacos e citocinas pró-inflamatórias na patogênese da miocardite aguda.^7-9^ A liberação excessiva de citocinas na COVID-19 já é um fato conhecido.^1-2^

Nossa hipótese é de que citocinas como TNF-α, IL-1, IL-6, IL-8, e IL-10, que são conhecidas por terem efeitos cardio-depressivos, e catecolaminas endógenas e exógenas, que desempenham papel importante na sepse, possam também desencadeiam o efeito cardio-depressivo na COVID-19. Além disso, consideramos que a miocardiopatia pode ser reversível ao remover-se as citocinas da circulação durante a recuperação. Estudos anteriores também demonstraram que a inibição da replicação viral mediada por tripsina e a downregulação de citocinas e metaloproteinases da matriz melhoraram significativamente as funções cardíacas de camundongos infectados pelo vírus da influenza A.^7-9^De acordo com esses achados, temos que identificar prontamente pacientes críticos e tratá-los o mais rápido possível, para evitar complicações fatais. Precisamos utilizar todos os tipos de ferramentas diagnósticas e opções de tratamento durante o seguimento. De maneira especial, a ETT pode ser a maneira menos dispendiosa e mais fácil de acompanhar esses pacientes. Entretanto, ainda não existe medicamento específico para o tratamento de pacientes com COVID-19. Com base na experiência do tratamento da SARS (Síndrome Respiratória Aguda Severa) e MERS (Síndrome Respiratória do Oriente Médio), alguns medicamentos como hidroxicloroquina, azitromicina, oseltamivir, lopinavir-ritonavir, remdesivir e favipiravir podem ter efeitos positivos em pacientes com COVID-19.^1^

Em conclusão, nossa paciente não apresentou miocardite, pois não houve aumento da troponina-I, mas acreditamos que ela possa sofrer miocardiopatia devido à liberação excessiva de citocinas. No nosso caso, a miocardiopatia e a COVID-19 foram tratadas com hidroxicloroquina, metilprednisolona, azitromicina e, finalmente, com favipiravir. No entanto, os efeitos curativos desses medicamentos ainda não foram comprovados e precisam de pesquisas adicionais.
