# Supplementary material for: Nova Pneumonia por Coronavírus e Miocardiopatia: Relato de Caso
Source: Arq Bras Cardiol. 2020 May 22;114(5):843–5. [Article in Portuguese] doi: 10.36660/abc.20200268 (PMC8387003; doi:10.36660/abc.20200268)
Supplement: Translation

# Translation: Novel Coronavirus Pneumonia and Cardiomyopathy: A Case Report

**Authors:** Mustafa Ahmet Huyut

**Affiliations:** 1 Turkey Yeni Yuzyil University – Cardiology, Istanbul – Turkey

**Keywords:** Cardiovascular Diseases/complications, Coronavirus, COVID-19, Myocarditis, Cardiomyopathies, Infectious Diseases, Acute Respiratory Syndrome, Pneumonia, Echocardiography/methods, Tomography, X-Ray Computed/methods, Hospitalization

A clustering of pneumonia was first recorded in Wuhan, Hubei, China in December 2019.^1^ A coronavirus has been established as the pathogen responsible for the disease, and has since been called Severe Acute Coronavirus Syndrome-2 (SARS-CoV-2). The disease triggered by SARS-CoV-2 is called COVID-19, which has spread worldwide since then. Figures tend to climb in Europe, and the extent of COVID-19 lethality cannot be correctly measured. In older patients, lethality appears to be particularly higher when compared to seasonal influenza.^2^ CT is very helpful in COVID-19 diagnosis.^3^ Also, TTE examination is a very important tool to asses LVEF. Previous studies reported that the real-time polymerase chain reaction (RT-PCR) was the current gold standard for COVID-19 diagnosis.^3^ But in some cases, the CT sensitivity is higher than that of RT-PCR.^4^ We report a confirmed case of COVID-19 pneumonia in a 59-year-old female. We found a mild decrease in LVEF without troponin-I elevation, which might be considered as cardiomyopathy due to the increased cytokine release in COVID-19. To the best of our knowledge, this is the first report in the literature demonstrating the association between TTE and CT images in COVID-19, and we found that the worsening in the TTE findings is in line with the progression of CT images.

## Case report

A 59-year-old female had had fever for 4 days, after catching a cold. One day before visiting the hospital, she had fever and cough, but no chest tightness, chest pain, chills, nausea and vomiting or diarrhea. She did not feel better after receiving antifebrile agents. Then, she was admitted to our outpatient clinic in BHT Clinic Tema Hospital. Four days before, the patient had had contact with her relative who had traveled from Europe. In her previous medical history, bariatric surgery had been performed 3 years before and she had still type II diabetes mellitus, hyperlipidemia, and hypertension as pre-existing conditions. She was hospitalized in our hospital on March/20/2020, and still had fever after admission, with the highest temperature of 39.5 °C, heart rate of 119 beats per minute; the electrocardiography was consistent with sinus tachycardia and QTc was calculated at 0.398 seconds, blood pressure at 94/60 mmHg, and conspicuous tachypnea with a respiratory rate of 24/min with sufficient oxygenation (95% ambient air saturation). The identification of 2019 novel coronavirus (2019-nCoV) in RT-PCR was positive from a throat swab. The risk of simultaneous contamination with other respiratory viruses and other pathogens were negative for the throat swab. The CT characteristics of the patient were similar to the case series reported by Pan et al.^5^ (Figure 1: A-B). Laboratory results showed leukopenia, with 4.1×10^9^/L, lymphopenia with 0.8×10^9^/L, an only slightly increased CRP level, with 18.4 mg/L and a low procalcitonin level, with 0.01 ng/mL. The patient underwent a TTE examination with a 3.5-MHz transducer (Vivid-7 GE Medical System, Horten, Norway). Examinations and measurements were performed according to the recommendations of the American Echocardiography Unit. Simpson’s method was used to calculate LVEF.^6^ At the admission, LVEF was calculated at 65%, with normal TTE findings.

**Figure 1:** – *Axial CT images. **(A-B)**: At admission, CT shows the bilateral presence of mild GGOs in the parenchyma. **(C-D)**: On day 6, a repeated CT was consistent with increasing expansion of the GGOs and progressed consolidations, which are called crazy paving consolidations. **(E-F)**: On day 12, a repeated CT showed that the previous consolidations and GGOs in both lungs were mostly absorbed, leaving fibrous lesions that may indicate residual organizing pneumonia. CT: computer tomography, GGOs: ground-glass opacities.*

The patient was isolated, and initiated nasal high flow therapy for respiratory insufficiency and treated with the antiviral drug (oseltamivir, 75mg/capsule, 1 capsule each time, twice a day, for 5-days), antibiotic (azithromycin, 500mg/tablet on the first day, and after that, 250mg/tablet, once a day for 4-days), antipyretic (paracetamol 1gr/100 mL, twice a day), mucolytic (N-acetylcysteine ampule, 300mg/3ml intravenous (IV), twice a day), anticoagulant (enoxaparin 4000 anti-Xa IU/0,4 mL, once a day), corticosteroid (methylprednisolone, 40mg intravenous (IV), once a day, for 5 days), proton-pump inhibitor (esomeprazole ampule, 40 mg IV, once a day), and antimalarial drug (hydroxychloroquine sulfate 200mg/tablet, 400 mg/tablet twice a day on the first day, and after that, 200mg/tablet twice a day, for 6-days). After 5 days of the treatment, the patient’s temperature dropped to normal and the symptoms disappeared. However, on day 6, a repeated CT was consistent with increasing expansion of the GGOs and progressed to which are called “crazy paving consolidations” (Figure 1: C-D). Moreover, LVEF was calculated at 52%, but the troponin-I level was still normal. Due to the CT scan results and TTE findings, we added favipiravir to the treatment (200mg/tablet on the first day, 1600mg/tablet twice a day, and 600mg/tablet, twice a day for 4 days) instead of oseltamivir. On day 12, a repeated CT showed that the previous consolidations and GGOs in both lungs were mostly absorbed, leaving some fibrous lesions that may indicate residual organizing pneumonia (Figure 1: E-F). Also, LVEF was calculated at 65% and a repeated RT-PCR was negative and the patient was discharged. No other follow-up CT examinations were performed.

The infection is primarily spread through respiratory droplets. Fever and dry cough are the primary clinical signs of COVID-19 in patients, accompanied by body aches or exhaustion and most patients were aged between 40 and 60 years-old. Also, in some cases headache and hemoptysis and diarrhea may occur. Moreover, severe patients may progress to ARDS and intubation may be required in certain patients.^1^ The clinical signs of COVID-19 are the same as those of normal upper respiratory tract infections, but the chest CT shows certain details.^5^ However, it is difficult to distinguish COVID-19 from other viral pneumonia based on CT findings alone. It is still necessary to clarify and define the epidemiological history, and it should be diagnosed by RT-PCR. Acute myocarditis is a documented risk of viral infections, such as influenza. Clinical presentation ranges from asymptomatic to fulminant myocarditis, which may contribute to severe hemodynamic instability.^7^ Previous autopsy-based studies on fatal cases showed that during the 1957 Asian influenza pandemic and during the Spanish influenza pandemic, 39.4% and 48% complication rates of focal to diffuse myocarditis were recorded, respectively.^8^ These deadly incidents of myocarditis showed both severe pneumonia and multiple organ involvement. As a consequence, myocarditis is expected to be a fatal risk in a pandemic influenza outbreak. Miura et al.^9^ also found a viral antigen in the myocardium with immunohistochemical staining of the autopsied heart.^9^Bowles et al.^10^ evaluated endomyocardial biopsy samples from 624 patients and objectively identified myocarditis utilizing PCR for different viral genes. Of the 239 samples tested positive for viral genes, adenovirus was found in 142 samples, enterovirus in 85 samples and influenza A in just five (0.8%) samples.^10^ Thus, although the pathogenesis of COVID-19-associated cardiomyopathy or myocarditis remains unclear, the literature suggests that endothelial dysfunction may have an important role in the pathogenesis of myocarditis and cardiomyopathy. Electron microscopic findings of the heart from a murine influenza myocarditis model showed many infiltrating lymphocytes directly attached to the cardiac myocytes, and proinflammatory cytokines in the pathogenesis of acute myocarditis.^7-9^ The over-release of cytokines in COVID-19 is already known.^1-2^

We hypothesized that cytokines such as TNF-α, IL-1, IL-6, IL-8, IL-10, which are known to have cardio-depressant effects, and endogenous and exogenous catecholamines, which play important role in sepsis, may also trigger the cardio-depressant effect in COVID-19. Also, we considered that the cardiomyopathy might be reversible by clearing cytokines from the circulation during recovery. Previous studies have also demonstrated that inhibiting trypsin-mediated viral replication and downregulation of matrix metalloproteinases and cytokines, significantly improved the cardiac functions of mice infected with influenza A virus.^7-9^ According to these findings, we have to promptly identify critically-ill patients and treat them as soon as possible, to avoid fatal complications. We need to use all kinds of diagnostic tools and treatment options during the follow-up. Especially, TTE may be the cheapest and the easiest way to follow-up these patients. However, there is still no specific drug for the treatment of COVID-19 patients. Based on the experience of the treatment of SARS and MERS, some drugs such as hydroxychloroquine, azithromycin, oseltamivir, lopinavir-ritonavir, remdesivir, and favipiravir might have positive effects on COVID-19 patients.^1^

In conclusion, our patient did not experience myocarditis, because there was no troponin-I increase, but we believe she might experience cardiomyopathy due to the over-release of cytokines. In our case, cardiomyopathy and COVID-19 were treated with hydroxychloroquine, methylprednisolone, azithromycin, and finally with favipiravir. However, the curative effects of these medications have not yet been proven, and still need further researches.
